# Effect of additives on the high-temperature performance of a sodium bis(oxalato)borate in triethyl phosphate electrolyte in sodium-ion batteries

**DOI:** 10.1038/s42004-025-01515-0

**Published:** 2025-04-26

**Authors:** Jonas Welch, Wessel W. A. van Ekeren, Jonas Mindemark, Reza Younesi

**Affiliations:** Department of Chemistry—Ångström Laboratory, Uppsala University, Uppsala, Sweden

**Keywords:** Batteries, Batteries

## Abstract

Sodium-ion batteries are a promising alternative to lithium-ion batteries due to their potential for lower cost and greater sustainability. However, achieving stable cycling performance, particularly at high mass-loadings and elevated temperatures, remains a challenge. The stable cycling of high mass-loading sodium-ion battery cells is here made possible by addition of prop-1-ene-1,3-sultone (PES) to a non-flammable and fluorine-free electrolyte solution of sodium bis(oxalato)borate (NaBOB) salt in triethyl phosphate (TEP). This study investigates the thermal stability and electrochemical performance of such electrolyte at 40 °C and 55 °C, contrasting their performance with base NaBOB in TEP with and without ethylene sulfate (DTD) additive and with a reference carbonate electrolyte of NaPF_6_ in ethylene carbonate:diethylene carbonate (EC:DEC). Nuclear Magnetic Resonance spectroscopy was used to reveal degradation products formed in the electrolyte following a 4-weeks storage at 55 °C. Results from galvanostatic cycling at 55 °C demonstrated comparable performance of NaBOB–TEP + PES electrolyte and the reference carbonate electrolyte. The internal cell resistance was initially lower when cells were cycled at 55 °C than at 40 °C for all studied electrolytes.

## Abstract (web summary)

Electrolyte additives are known to enhance the performance and life cycle of sodium-ion batteries at room temperature, but studies focusing on high-temperature conditions are lacking. Here, the authors report on the effect of additives on the thermal stability and performance of a non-flammable and fluorine-free electrolyte solution at 40 °C and 55 °C.

## Introduction

To limit global warming, batteries are a key technology that can be used to greatly reduce greenhouse gas emissions from transportation and electricity storage^1,2^. Lithium-ion batteries (LIB) are currently used in different applications as technology development and economies of scale have brought down the cost and increased energy density to attractive levels^3^. However, the rapidly growing adaption of LIB raises concerns regarding availability of raw materials used, such as lithium, cobalt, nickel, copper, and natural graphite^3^.

Sodium-ion batteries (SIB) are an alternative technology that share a number of qualities with LIB, but can be produced using fewer scarce resources and possibly lower cost^4,5^. Also, the higher safety of SIB compared to LIB is among the described advantages^6^. As negative electrode in SIB, hard carbon is commonly used, combining a stable electrochemical cycling with low operating potential and reasonable intercalation capacity of sodium ions^7^. As positive electrode active material, a range of materials have been proposed, such as layered transition metal oxides^8^, polyanionic species^9^ and Prussian blue analogues^10^. Prussian white (Na_2–x_Fe[Fe(CN)_6_]_1–y_·zH_2_O) is a material with attractive properties, such as high theoretical sodium storage capacity, relatively high electrode potential and naturally abundant precursor elements^10,11^. It has also been shown to tolerate high temperature battery operation^12^.

As in commercial LIBs, an electrolyte solution comprising sodium hexafluorophosphate (NaPF_6_) electrolyte salt dissolved in at least two carbonate-based solvents is often used^5^. However, the toxicity and cost of NaPF_6_ and the flammability of carbonate solvents motivates research into alternative electrolytes^13,14^. Moreover, the low chemical stability of LiPF_6_ and NaPF_6_ at high storage or battery operation temperatures is also a known problem leading to reduced battery lifetime^15–17^.

Increasing the cycle life at elevated temperatures is an important, but often not prioritized, aspect of battery research and development^18,19^. If batteries are more resilient to changes in temperature conditions, both in terms of safety and cycling performance, less complex and costly cooling and heating systems need to be integrated with battery cells in e.g., electric vehicles^18,19^. This can also increase the practical energy density, as thermal management systems require a lot of space and weight. Electrolytes based on ionic liquids are sometimes proposed as thermally stable electrolytes, with stability up to 300 °C and beyond^20^. However, ionic liquids are expensive and rarely used in battery electrolytes for this reason. Zheng et al. studied degradation of LiFP_6_ in EC:EMC (3:7, v/v) stored in different temperatures, and identified reaction products from degradation of PF_6_^−^ initiated by traces of HF and water or short alcohols and proposed reaction mechanisms for their formation. To alleviate the thermal degradation, an alternative electrolyte solution based on lithium (fluorosulfonyl)(n-nonafluorobutanesulfonyl)imide (LiFNFSI) salt was proposed^21^. For SIB, Barnes et al. studied thermal degradation based on NaPF_6_ in EC:EMC or EC:DMC in room temperature and 52 °C and with different moisture concentrations. There, degradation products of PF_6_^−^ were also identified^22^.

Electrolyte additives have been extensively utilized to enhance the performance and cycle life of LIBs and SIBs^23,24^. Among these additives, sulfur-containing compounds like prop-1-ene-1,3-sultone (PES) and 1,3,2-dioxathiolane 2,2-dioxide (DTD or ethylene sulfate) stand out for their ability to form protective layers on LIB anode surfaces^25^. For instance, DTD has demonstrated the capability to improve coulombic efficiency, capacity retention, and low-temperature performance^26,27^. Additionally, it suppresses graphite exfoliation in propylene carbonate (PC)-based electrolytes^26^. However, while DTD effectively reduces gas release during high-temperature storage^25^, this advantage does not extend to room temperature^23^. Furthermore, SEI layers formed exclusively by DTD are insufficiently stable for prolonged cycling of NMC111-graphite cells at elevated temperatures (55 °C) when used with carbonate-based electrolytes^28^. PES is another promising additive known for enhancing cycling performance in LIBs, with an optimal concentration of ~3 wt%^29^. Compared to the widely used vinylene carbonate (VC) additive, PES significantly reduces gas formation at elevated temperatures^30^. PES-containing electrolytes are capable of operating across a wide temperature range; however, cell resistance limits their performance at lower temperatures^31^. PES forms stable organic and inorganic sulfites in the SEI playing a key role in capacity retention^29^ and enabling high-voltage cycling of NMC-graphite cells^31^. Additionally, PES prevents graphite exfoliation even under high-temperature conditions, such as 70 °C^31^.

While research into improving SIB systems is gaining momentum, studies on electrolyte additives for SIBs remain relatively limited. Most investigations focus on room-temperature performance and commonly used additives like fluoroethylene carbonate (FEC) and VC^24^. Yan et al. combined the four electrolyte additives VC, succinonitrile (SN), 1,3-propane sultone (PS), and sodium difluoro(oxalate)borate (NaDFOB) to stabilize cycling at 55 °C of Na_3_V_2_(PO_4_)_2_F_3_ (NVPF)—Hard carbon full cells^32^.

We have previously reported NaBOB salt dissolved in TEP solvent as a non-flammable, non-toxic electrolyte solution for SIBs^33–35^. NaBOB has a limited solubility in common battery electrolyte solvents, and the concentration in TEP is limited to below 0.38 M. The performance of such an electrolyte in full-cell SIBs was further optimized by electrolyte additives. The results showed that PES and DTD were the most efficient additives in improving cycling performance, reaching a capacity retention of 80% after 450 cycles^36^.

This study investigates electrolyte stability and cycling performance of full-cell SIBs at elevated temperatures of 40 °C and 55 °C using NaBOB–TEP electrolyte with the aforementioned additives. 1 M NaPF_6_ in EC:DEC 1:1 v/v is here used as a standard reference electrolyte solution. Nuclear magnetic resonance (NMR) and visual inspection are used to investigate chemical changes in the electrolyte solutions upon storage at elevated temperatures. Electrochemical characterization including galvanostatic cycling and intermittent current interruption (ICI) technique are used to evaluate cell performance and internal resistance. Pressure analysis is used to investigate gas formation during formation cycling. The investigations show that the DTD additive is not thermally stable in the TEP-based electrolyte, as NMR analysis detects reaction products between DTD and TEP. In contrast, the PES additive enhances the cycling performance of the NaBOB–TEP electrolyte while also reducing total cell resistance and minimizing gas evolution during early cycling.

## Results and discussion

### Thermal stability of electrolyte solutions

**Fig. 1:**
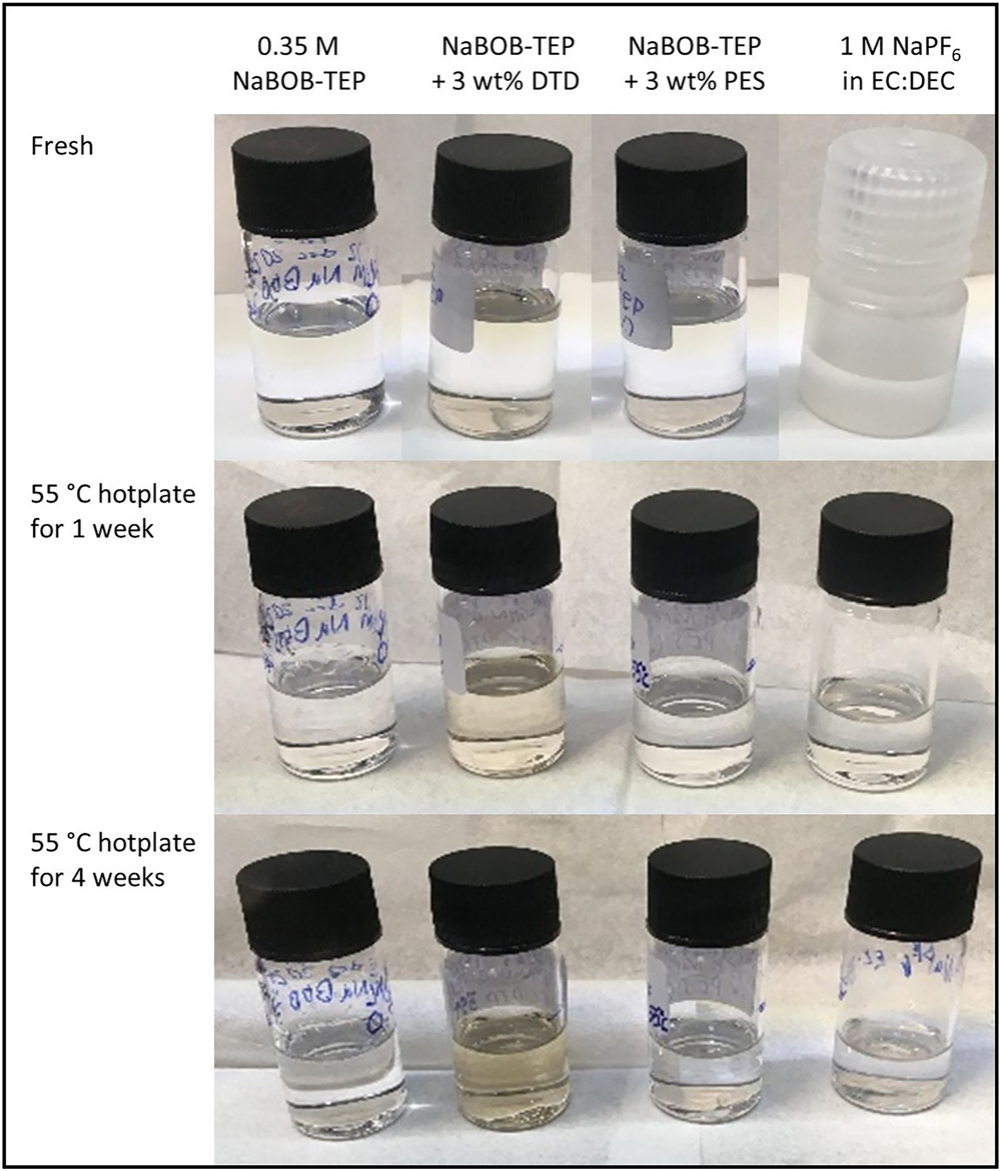
Visual color change after storage at elevated temperature. Digital photos of the freshly prepared electrolyte solutions and electrolyte solutions stored at 55 °C for 1 week or 4 weeks. After 1 week and 4 weeks small samples were taken from each vial for NMR spectroscopy analysis, which showed that the visible color change seen in the figure could be explained by a reaction between ethylene sulfate (DTD) and triethyl phosphate (TEP). Note that the sodium hexafluorophosphate (NaPF_6_) in ethylene carbonate:diethylene carbonate (EC:DEC) electrolyte solution was stored in a polypropylene plastic vial during hot storage, it was only transferred to a glass vial for the photo.

To investigate thermal stability of the studied electrolytes, specific amounts of each electrolyte was subjected to 55 °C for one- and four-week time periods. As shown in Fig. 1, NaBOB–TEP electrolyte with DTD additive exhibited a color change to a weakly yellow/orange color progressively during storage. Conversely, no clear color changes were observed in any of the other electrolyte solutions.

The fresh and aged electrolytes stored for 1 and 4 weeks of storage at 55 °C were further analyzed using in ^1^H, ^13^C, ^19^F and ^31^P NMR spectroscopy (Figs. S1–S13). ^19^F NMR spectra disclose that traces of breakdown products of PF_6_^−^ form in the NaPF_6_ in EC:DEC form after storage at 55 °C (Fig. 2a). Analogous to the interpretation made by Zheng et al., Barnes et al., Wilken et al., and Campion et al., the peaks appearing at around 80 and 82 ppm could correspond to difluorophosphoric acid (O=PF_2_(OH) or PO_2_F_2_^−^)^15,21,22,37^.

**Fig. 2:**
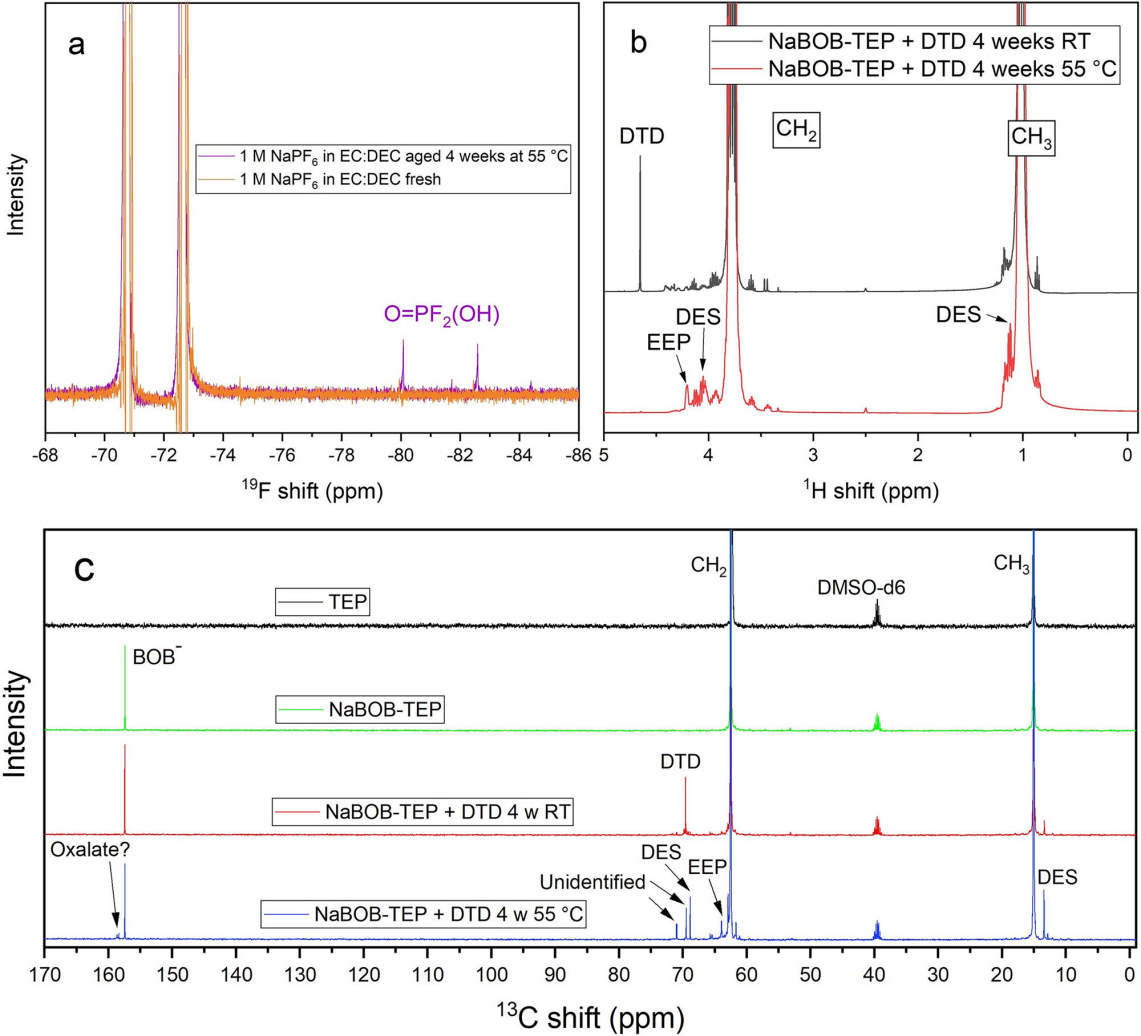
NMR spectra showing chemical degradation of two of the used electrolytes. **a** ^19^F NMR before (orange) and after (purple) storage of 1 M NaPF_6_ in EC:DEC for 4 weeks at 55 °C. Peaks at 80 and 82 ppm are identified as reaction products of PF_6_^−^, likely difluorophosphoric acid (O=PF_2_(OH). **b** ^1^H NMR spectra of 0.35 M NaBOB in TEP + 3 wt% DTD stored 4 weeks in room temperature (black, top) and 4 weeks at 55 °C (red, bottom). **c** ^13^C NMR spectra of TEP (black), 0.35 M NaBOB in TEP (green), 0.35 M NaBOB in TEP + 3 wt% DTD stored for 4 weeks at room temperature (red), and 0.35 M NaBOB in TEP + 3 wt% DTD stored 4 weeks at 55 °C (blue), from top to bottom, respectively.

The NaBOB–TEP + DTD electrolyte, which was visibly discolored after storage on the hotplate and also to a smaller degree after storage at room temperature, fully lost its characteristic DTD peak in the ^1^H spectrum (Fig. 2b) after storage at 55 °C for 4 weeks compared to four weeks at room temperature. This indicates that DTD decomposes at these conditions. The peaks detected at around 4.05 and 4.2 ppm are related to degradation products of DTD. Also, in the ^13^C spectrum (Fig. 2c), the DTD peak at 69.55 ppm diminishes and new peaks appeared at 70.97 (d), 69.46 (s), 68.82 (s), 63.90 (d) and 62.90 (d) and 61.65 (d) ppm (as can be more clearly seen in Fig. S7), indicating degradation of DTD at 55 °C. Some of these peaks are already visible in the sample from the electrolyte solution aged 4 weeks in room temperature, although in much smaller scale. This means that DTD starts to degrade already at room temperature, which is consistent with the visible color change. The ^1^H, ^13^C, ^19^F, and ^31^P NMR spectra for NaBOB–TEP and NaBOB–TEP + PES electrolytes were remained unchanged (see Figs. S1–2, S5–6), and were thus chemically stable at 55 °C. The peaks detected around 3.45 ppm in the ^1^H spectra and at 2.5 ppm in ^31^P spectra for TEP-based electrolytes already before thermal storage (Figs. S1 – S3 and Figs. S10–12, respectively) are believed to come from solvent impurities.

**Fig. 3:**
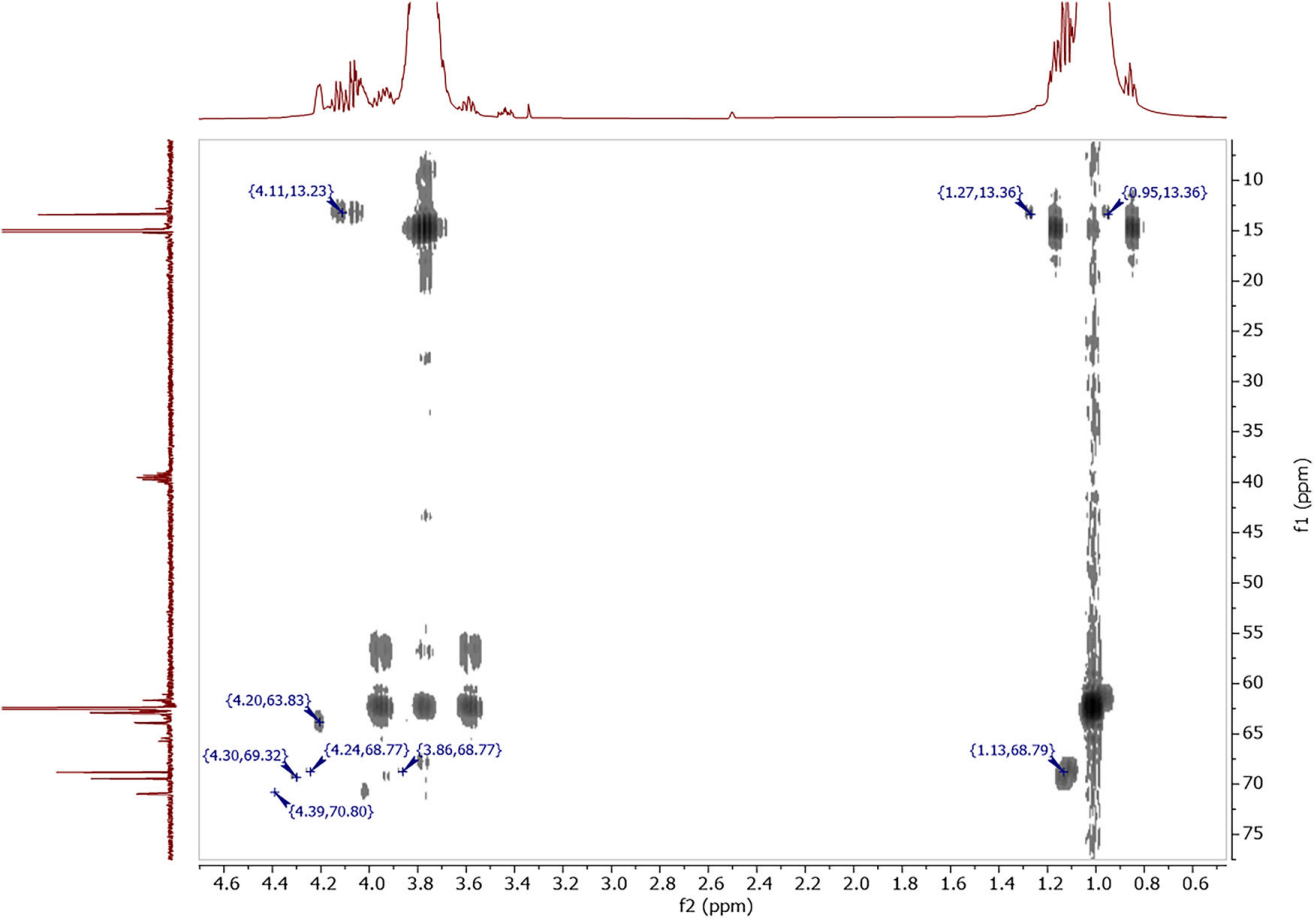
Evidence of reaction products of DTD and TEP. Heteronuclear Multiple Bond Coherence (HMBC) 2D NMR based on correlations between ^1^H (horizontal) and ^13^C (vertical) nuclei in the NaBOB–TEP + DTD electrolyte. Some of the peaks related to degradation products of DTD show HMBC correlation. Those are labeled in the figure and explained in Table 1.

To identify the degradation products in the NaBOB–TEP + DTD electrolyte, 2D NMR experiments were performed. First, a Heteronuclear Single Quantum Correlation (HSQC) NMR was made, but no peaks related to degradation products were detected (Fig. S14). Then, a Heteronuclear Multiple Bond Coherence (HMBC) NMR was made (Fig. 3), which shows correlations between ^1^H and ^13^C nuclei placed at least two atomic bonds apart in the molecule, as well as ^1^H correlations with ^13^C satellite peaks (one bond apart). Predicted ^1^H and ^13^C shifts in spectra from the Spectral Database for Organic Compounds and from a study by Xiao et al. were used to correlate the HMBC peak positions labeled in Fig. 3 to bonding environments within the degradation product molecules^38,39^. The suspected reaction products and the corresponding HMBC peak positions are further described in Table 1.

**Table 1:** Identified degradation products in the NaBOB–TEP + DTD electrolyte

^1^H peak	^13^C peak	Compound	Correlating nuclei in HMBC
1.13	68.79	Diethyl sulfate	Proton on carbon with carbon
4.11	13.23	Diethyl sulfate	Proton on carbon with carbon
1.27, 0.95	13.36	Diethyl sulfate	Carbon with proton on carbon (^13^C satellite peak)
4.24, 3.86	68.77	Diethyl sulfate	Carbon with proton on carbon (^13^C satellite peak)
4.20	63.83	Ethyl ethylene phosphate	Proton on carbon with other carbon
4.30	69.32	Unidentified product 1	
4.39	70.80	Unidentified product 2	

**Scheme 1:**
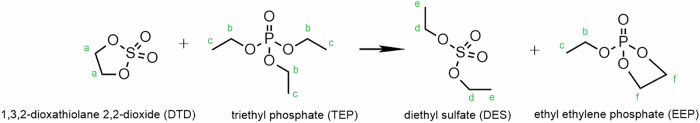
**Chemical reaction of DTD with TEP**. Transesterification reaction degrading the NaBOB–TEP + DTD electrolyte solution. Chemically different carbon atoms have been marked with letters a–e in green.

Letters in green are referring to chemically different carbon atoms, indicated in Scheme 1.

The degradation products, diethyl sulfate and 2-Ethoxy-1,3,2-dioxaphospholane 2-Oxide (ethyl ethylene phosphate), are hypothesized to be formed via a transesterification reaction, as depicted in Scheme 1, where the chemically different carbon atoms are also indicated.

### Galvanostatic cycling performance

**Fig. 4:**
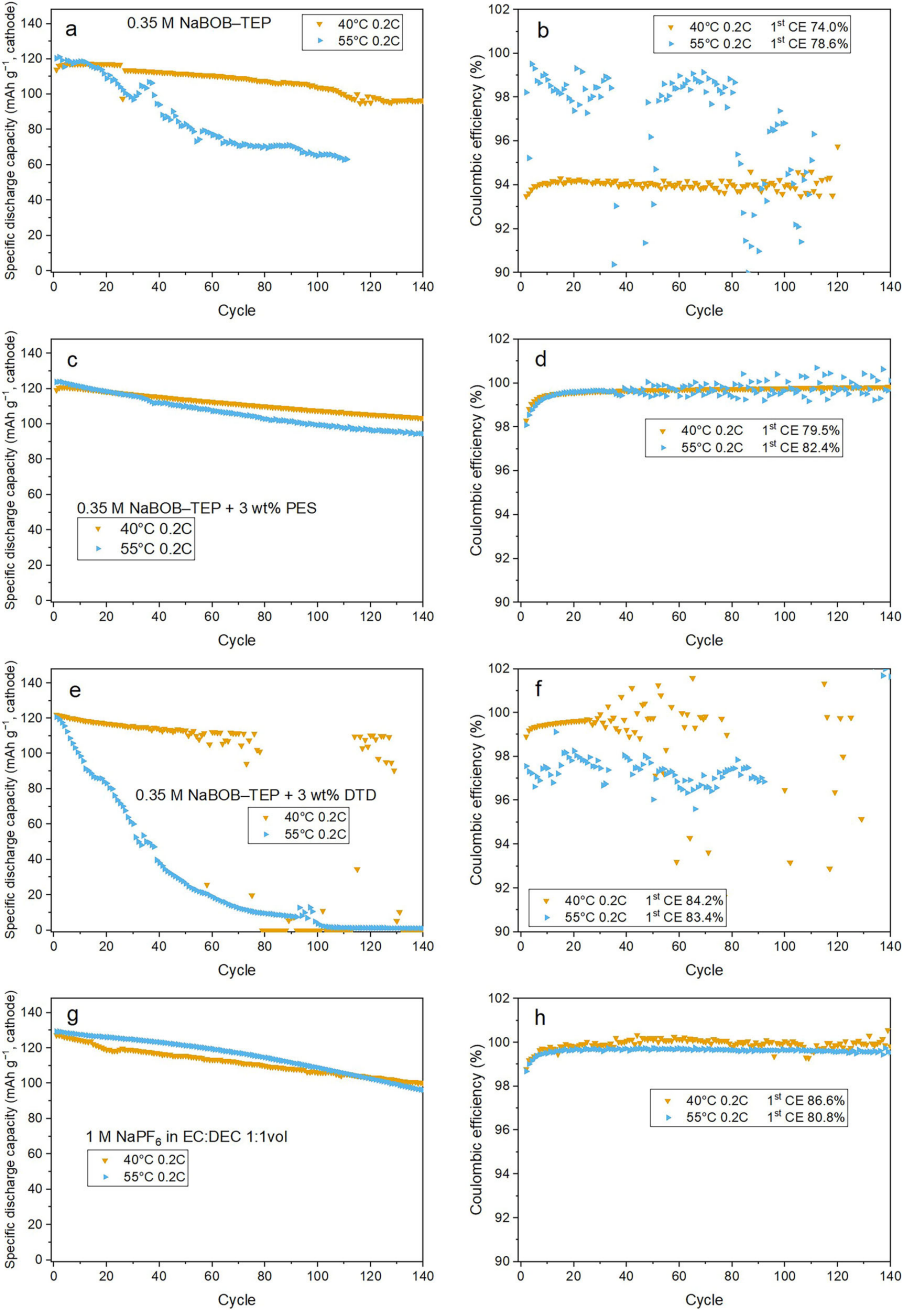
Capacity retention in galvanostatic cycling. Galvanostatic cycling of Prussian white – Hard carbon sodium-ion full cells at 40 °C (orange triangles) or 55 °C (blue triangles) in the left column and coulombic efficiency of the same cells in the right column. Used electrolytes were in **a** and **b** 0.35 M NaBOB in TEP, in **c** and **d** 0.35 M NaBOB in TEP + 3 wt% PES, in **e** and **f** 0.35 M NaBOB in TEP + 3 wt% DTD and in **g** and **h** 1 M NaPF_6_ in EC:DEC 1:1 by volume.

Figure 4 shows discharge capacities of full-cells galvanostatically cycled at elevated temperatures of 40 °C and 55 °C at a cycling rate of 0.2 C using different electrolytes studied in this work. The cells using 0.35 M NaBOB–TEP without any additives exhibited substantial capacity fading at both tested temperatures, although some improvement is observed compared to the previous cycling conducted at room temperature^36^. This is consistent with our previous study, where 0.35 M NaBOB–TEP showed rapid fading in cells with high mass-loading electrodes (12 mg cm^−2^) unless a specific formation cycling protocol is applied^40^. The addition of PES to NaBOB–TEP electrolyte led to large improvements in cycling stability as the cells cycled at both temperatures showed smaller fading, although the increased temperature predictably impacted capacity fading to some degree (see Fig. 4c, d and S15). This increase in capacity fading can be partly alleviated by the use of a faster C-rate (see Fig. S15), which will decrease the time during which unwanted side-reactions can occur. The likely cause for the high thermal stability is that PES forms a sulfite-rich SEI which is effectively passivating the anode also at elevated temperature, preventing electrolyte degradation and gas formation^41^. The cells with the DTD additive suffered from fast capacity degradation at 55 °C, whereas degradation was initially low at 40 °C after which cycling became unreliable (see Fig. 4e, f). This is likely related to the thermal instability of the DTD as discussed above, leading to a loss of DTD in the electrolyte and thus its ability to reform lost SEI during cycling. Similarly, Madec et al. have shown that the use of DTD as a single additive in LIB cells using a LiPF_6_ electrolyte produce an unstable SEI on graphite negative electrodes leading to accelerated capacity fading after 150 cycles^28^.

Figure 4g, h present the cycling results of the reference cells using the carbonate electrolyte. The cells exhibited a relatively higher first cycle CE, with the capacity fading of the cell cycled at 40 °C comparable to those with PES. However, the cell cycled at 55 °C showed an accelerated degradation after nearly 60 cycles. The addition of PES and DTD to the NaPF_6_ in EC:DEC electrolyte showed detrimental effect on the long-term cycling performance of these cells with carbonate-based electrolytes, see Fig. S16, and are thus not suitable as high-temperature stable electrolytes.

### Internal cell resistance

**Fig. 5:**
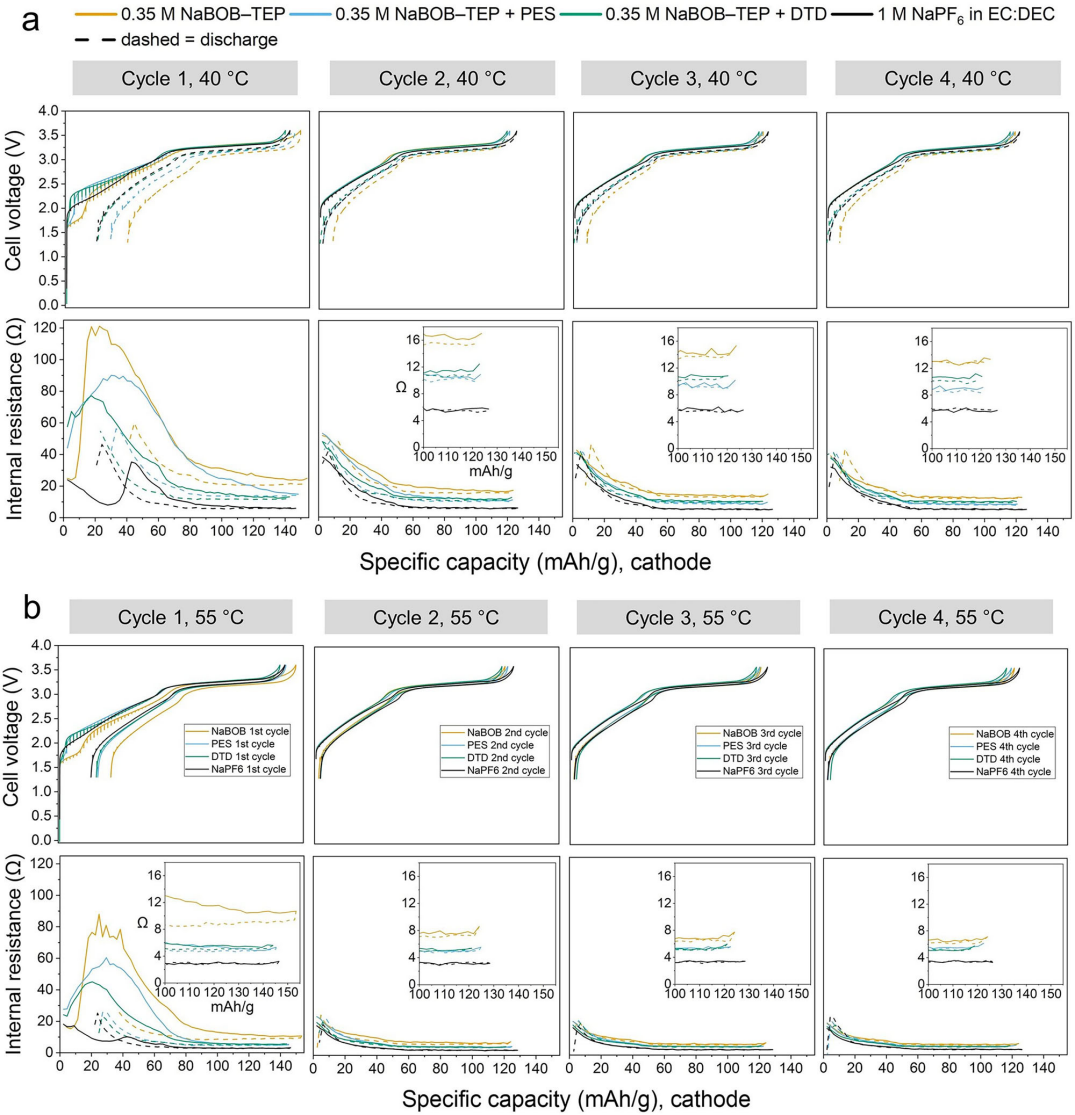
Voltage profiles and internal resistance in cells cycled at elevated temperature. Voltage profiles as well as internal cell resistance measured with the ICI method for Prussian white—Hard carbon full-cells with four different electrolytes (0.35 M NaBOB–TEP (orange), 0.35 M NaBOB–TEP + 3 wt% PES (blue), 0.35 M NaBOB–TEP + 3 wt% DTD (green) and 1 M NaPF6 in EC:DEC (black)), cycled at 0.2 C at **a** 40 °C and **b** 55 °C temperatures.

To investigate internal cell resistance during cycling, the ICI method was used (Fig. 5). During the initial stages of the first charge, the cells based on NaBOB–TEP with and without PES and DTD additives experienced high resistance, attributed to SEI formation (Fig. 5a). Conversely, cells employing NaPF_6_ in EC:DEC demonstrated comparatively lower resistance. Instead, the resistance slightly increases at around 40 mAh g^−1^ during the first charge (Fig. 5a and S17). Throughout subsequent cycles, all the cells exhibited a consistent pattern during both charge and discharge, consistently peaking at low states of charge. This phenomenon may be linked to low Na^+^ mobility in the cathode active material when the Na content is high. It should be noted that among other parameters, the applied pressure to the pouch cells could have a large influence on the cell resistance. A higher controlled pressure may favor the cycling performance and lower the resistance in cells. To investigate the validity of the resistance calculations, a sample of resistance data points were scrutinized. Linear regression of the voltage as a function of the square root of time shows reasonably good fits, as can be seen in Fig. S18.

### Rate capability

**Fig. 6:**
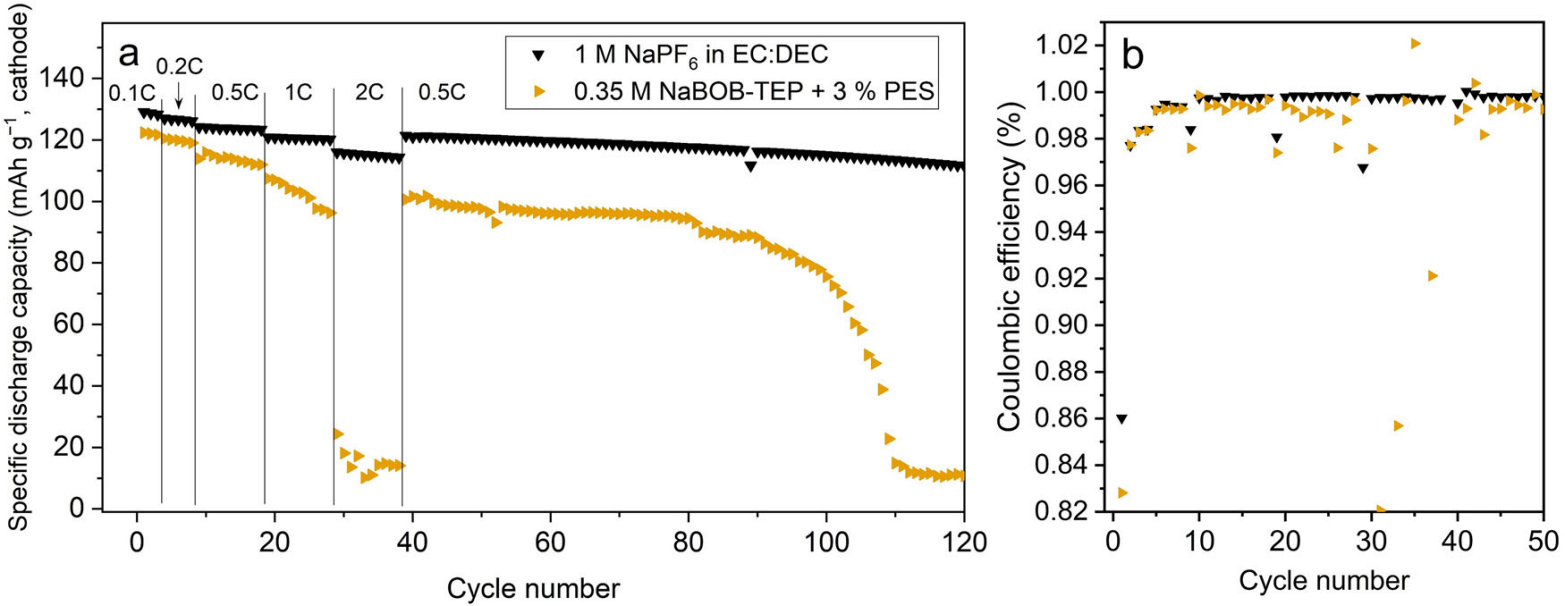
Galvanostatic cycling at increasing rate. Rate test at 55 °C for Prussian white – Hard carbon full cells using 0.35 M NaBOB–TEP + 3 wt% PES (orange triangles) and 1 M NaPF_6_ in EC:DEC (black triangles) electrolytes, respectively. Specific capacity (**a**) and coulombic efficiency (**b**).

A rate-test was performed for the NaPF_6_–EC:DEC and NaBOB–TEP + PES electrolytes at 55 °C, as the higher temperature enables higher ionic conductivity and potentially lower overall resistance. The results in Fig. 6 show that the cell with carbonate-based electrolyte outperformed the cell with 0.35 M NaBOB–TEP + PES at high C-rates as the latter suffered from major capacity fading at 2 C. The cause of this could be a result of the relatively lower ionic conductivity of NaBOB–TEP electrolyte, resulting in mostly active material close to the macro surface of the electrodes contributing with sodium ions. Alternatively, it could stem from a high-impedance SEI layer, as evidenced by the nearly doubled cell resistance measured via ICI (see Fig. 5).

### Capacity retention after extended cycling pauses

**Fig. 7:**
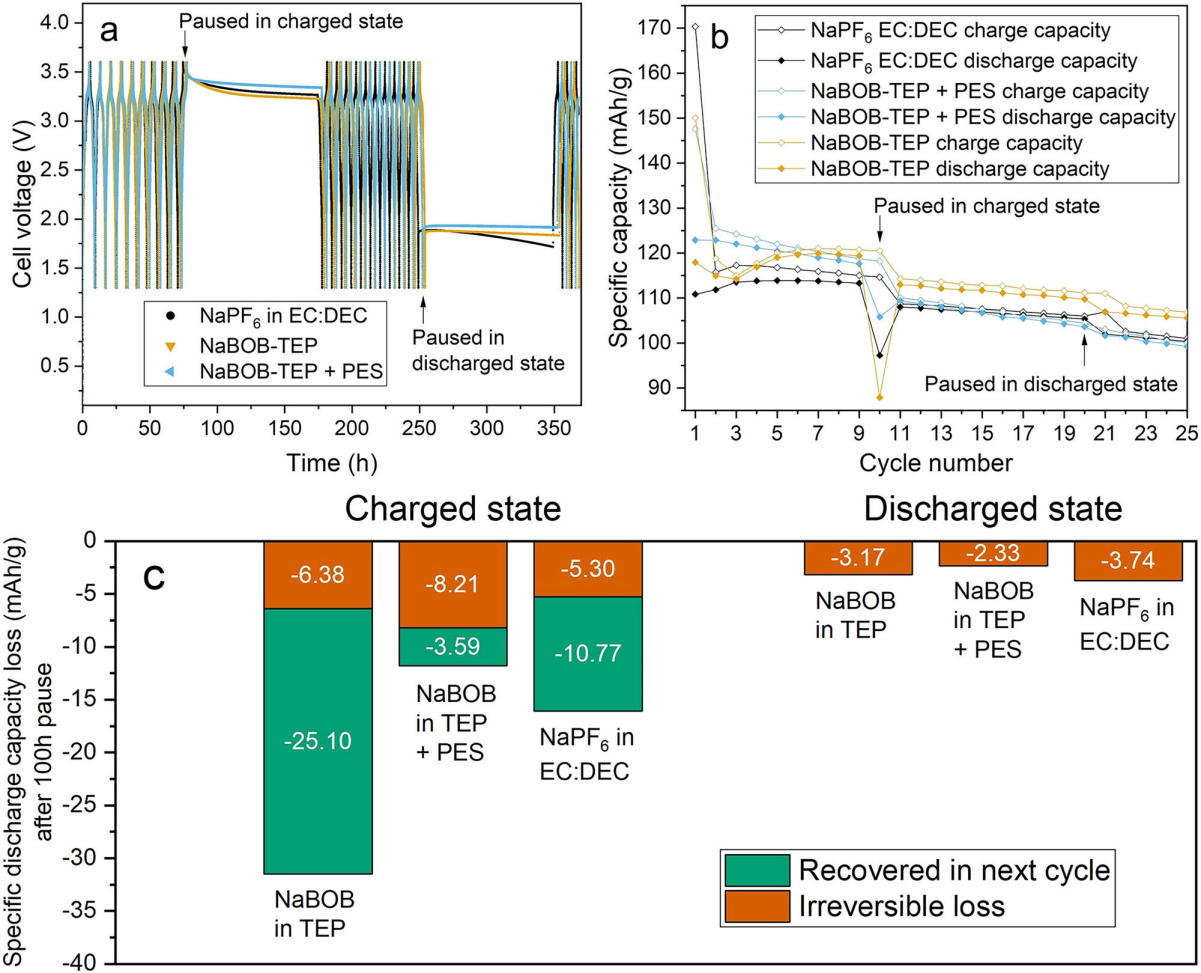
Capacity loss after extended cycling pauses. Galvanostatic cycling combined with extended relaxation test of Prussian white—Hard carbon full-cells using the three electrolytes of 0.35 M NaBOB in TEP (orange triangles or diamonds), 0.35 M NaBOB in TEP + 3 wt% PES (blue triangles or diamonds) or 1 M NaPF_6_ in EC:DEC 1:1 (black circles or diamonds). **a** Overview of the voltage profiles. The cells were cycled at 55 °C using 0.2 C rate, and relaxed for 100 h after charge number 10 and relaxed for 100 h after discharge number 20. **b** Corresponding charge and discharge capacities for the same cells. Open diamonds for charge capacities and filled diamonds for discharge capacities. **c** Calculated losses in the first discharge following the pause compared to the last discharge before the pause, for pauses at charged and discharged states, respectively. Irreversible loss (red) remains also in the second discharge after the pause whereas the capacity which is recovered in the next cycle (green) is the reversible loss that is only seen in the first discharge after the pause.

To study the self-discharge, extended pause/relaxation test was carried out where cells were cycled at 0.2 C at 55 °C and were then stored at open circuit potential for 100 h after the 10th charge and the 20th discharge (Fig. 7a). The capacity values for the subsequent two discharges after the pause (Fig. 7b) was used to calculate the losses related to degradation taking place in the pause (total loss), losses that can be recouped in the second discharge after the pause (reversible loss) and the difference between the two (irreversible loss). The results overall (Fig. 7c) show that the losses for all three electrolytes are substantial, which is consistent with earlier studies on ageing of SIBs^42^. Furthermore, it is also evident that the losses are greater after a pause at charged state, of which the majority is recoverable (i.e., reversible losses) for NaPF_6_ in EC:DEC electrolyte and NaBOB–TEP without additive, whereas for the NaBOB–TEP with PES additive, the irreversible losses dominated. The reversible losses during the pause may be attributed to a redox shuttling mechanism, where guest molecules present in the electrolyte carry charge by diffusion between the anode and cathode while repeatedly undergoing reduction and oxidation. Sloop et al. linked this phenomenon to the shuttling of CO₂ formed by reduction of EC^41^. Melin et al. has showed that CO₂ is also produced from the reduction of LiBOB, and thus could also potentially contributing to the effect^43^. The irreversible losses are likely caused by SEI dissolution and subsequent re-formation, which consumes part of the sodium inventory. In the case of PES-containing electrolyte, this effect may be exacerbated by a more organic SEI that is more prone to dissolution^31,44^ compared to the more inorganic SEI formed by NaBOB and TEP^36^. A pause at the discharged state did not result in reversible losses, and irreversible losses were slightly lower compared to those at the charged state for all three electrolytes. The voltage curves seen in Fig. 7a show that NaBOB–TEP electrolyte without additive follow a self-discharge curve similar to that of NaPF_6_–EC:DEC but with a faster overall self-discharge, which is also evident in the lower discharge capacity directly following the pause. On the other hand, the cell with the electrolyte with PES additive experience a faster drop below 3.45 V than the two other electrolytes, but then stabilizes and show a much lower decrease in voltage, ending the pause at a higher value of 3.34 V compared to 3.27 V for NaPF_6_ in EC:DEC and 3.23 V (corresponding to the higher plateau of Prussian white) for NaBOB–TEP without additive. This phenomenon is also evident in the much lower reversible losses for the electrolyte with PES additive (Fig. 7c). From a user perspective, a cell with low self-discharge is useful, since you have more useful capacity after a pause, however from a state of health perspective, the irreversible losses need to be considered. In that regard, the losses are comparable between the studied electrolytes, and only marginally higher for the NaBOB–TEP electrolyte with PES additive.

### Gas pressure evolution in early cycling

**Fig. 8:**
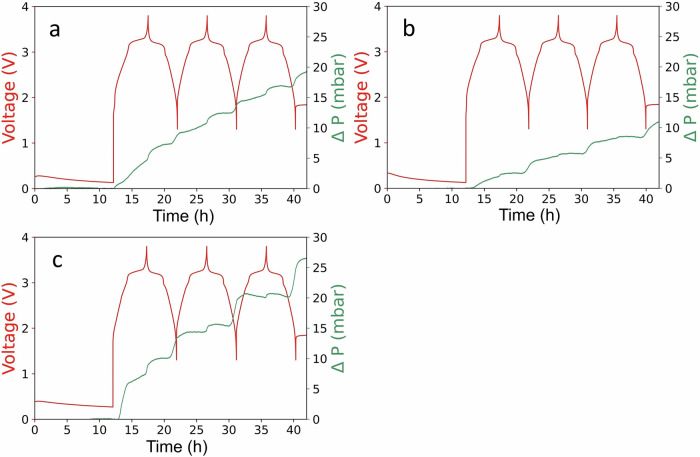
Pressure evolution in first cycles. Pressure analysis (green lines, right-hand scale) of full-cells cycled at 55 °C at 0.2 C rate using **a** 0.35 M NaBOB in TEP, **b** 0.35 M NaBOB in TEP + 3 wt% PES and **c** 1 M NaPF_6_ in EC:DEC electrolyte solutions, as well as voltage profiles for the same cells (red lines, left-hand scale). Note that there is a natural leakage of the pressure cells specified to maximum 0.3 mbar h^−1^ by the supplier, and that the relative pressures have been corrected to reflect an estimate of the pressure evolved from the cell components during the galvanostatic cycling. The original pressure data is available in Fig. S19.

The pressure buildup in cells during initial three cycles at 55 °C was measured, as shown in Fig. 8. The cells with NaBOB in TEP or with NaPF_6_ in EC:DEC electrolytes showed a substantial increase in the cell pressure, i.e. 20 mbar and 25 mbar respectively (Fig. 8a, c). However, the pressure increase in the cell using NaBOB–TEP + PES electrolyte is considerably lower (i.e. around 10 mbar), indicating that the surface of the negative electrode is effectively passivated using PES additive (Fig. 8b). It should be noted that these cells experience a leakage specified to maximum 0.3 mbar h^−1^ by the supplier, and thus the figures below have been corrected for this leakage by an addition of a linear component to the data corresponding to the slope of the measured pressure during the 2 h directly before the initiation of the galvanostatic cycling. The original pressure data is available in Fig. S19. These results are consistent with literature for LIB cells, where Xia et al. showed that PES considerably lowers gas volume formed in Li(Ni_1/3_Mn_1/3_Co_1/3_)O_2_/graphite pouch cells using a standard LiPF_6_ electrolyte upon cycling at 40 °C and storage at 60 °C, compared to the VC additive or no additive^30^.

## Conclusions

The thermal stability of NaBOB–TEP electrolytes with PES or DTD additives as well as a reference carbonate electrolyte based on NaPF_6_ in EC:DEC was studied through storage at elevated temperature of 55 °C. NMR spectroscopy results revealed partial degradation of NaPF_6_ likely forming difluorophosphoric acid (O=PF_2_(OH), and full degradation of DTD in a transesterification reaction with TEP solvent forming diethyl sulfate and ethyl ethylene phosphate. The degradation of DTD in the NaBOB–TEP electrolyte solution starts already at room temperature, discoloring the electrolyte. However, NaBOB–TEP electrolyte with and without PES additive remained stable after 4 weeks storage at 55 °C.

The electrochemical performance of Prussian white–hard carbon full-cells was investigated at 40 °C and 55 °C under different cycling protocols, including galvanostatic cycling with and without extended relaxation time and different cycling rates. 0.35 M NaBOB in TEP with 3 wt% PES electrolyte solution showed promising results, obtaining stable cycling at 55 °C at 0.2 C rate and reaching comparable capacity retention after 140 cycles as 1 M NaPF_6_ in EC:DEC at the same condition. The NaFP_6_ electrolyte on the other hand experienced an acceleration in capacity fade after around 100 cycles. Rate capability tests at 55 °C exhibited inferior performance for NaBOB–TEP + PES electrolyte compared to NaPF_6_ in EC:DEC, which could be mainly due to the relatively lower ionic conductivity of this electrolyte and/or higher SEI-layer impedance, as is implied by the higher cell resistance.

PES additives decreased the amount of gas formed during the initial cycles and thus the cell pressure was lower in the cell with NaBOB–TEP + PES electrolyte compared to the cells with NaBOB–TEP or NaPF_6_ in EC:DEC. The cell resistance formed during the 1st charge was higher in the cells with NaBOB in TEP with and without additives, compared to the reference cell with NaPF_6_ in EC:DEC. However, that decreased to smaller values similar to the resistance in NaPF_6_ in EC:DEC cells from the 1st discharge, and remained more or less the same in the following cycles. All this together suggest that the ratio of solid to gas species formed during the 1st cell charge is higher in NaBOB–TEP + PES electrolyte compared to that in NaBOB–TEP or NaPF_6_ in EC:DEC.

Furthermore, pause tests show a lower self-discharge rate of cells with NaBOB–TEP + PES electrolyte compared to NaPF_6_ EC:DEC, which is evident by the slower voltage decay and lower reversible capacity loss when paused in the charged state. However, the irreversible loss is somewhat higher than for the reference carbonate electrolyte.

Overall, NaBOB–TEP + 3 wt% PES as a non-flammable and fluorine-free electrolyte is a promising alternative to flammable carbonate-based electrolytes, with potential use in commercial SIB suitable for applications with higher operating temperatures.

## Methods

### Materials, electrolyte preparation and thermal stability test

Electrolytes were prepared from NaBOB synthesized and purified, according to the method described earlier^38^. NaPF_6_ (Stella®, 99%) was dried at 120 °C in a vacuum oven before use. PES (TCI chemicals®, ≥99%) and DTD (TCI chemicals®, ≥98%) were used as electrolyte additives. TEP (Sigma-Aldrich®, ≥99.8%) as well as EC:DEC 1:1 (Gotion®) was kept over molecular sieves at least 1 day before electrolyte preparation. NaBOB–TEP electrolyte solutions for electrochemical experiments were prepared volumetrically in glass vials, whereas the NaPF_6_ in EC:DEC electrolyte solutions was prepared and stored in aluminum vials. All electrolytes were prepared and stored in an argon-filled glovebox with water and moisture levels below 1 ppm. Moisture content in electrolyte solutions was 14.5 ppm for NaPF_6_ in EC:DEC and <34 ppm for NaBOB in TEP, measured with Karl Fisher titration.

Other separate batches of electrolyte solutions, specifically prepared for the storage test at elevated temperature, were treated as follows. NaBOB–TEP, NaBOB–TEP + PES and NaBOB–TEP + DTD electrolyte solutions were all prepared and stored in glass vials. NaPF_6_ in EC:DEC was prepared in a glass vial, poured to an aluminum vial and stored for 20 days in room temperature, and then poured to a polypropylene plastic vial before elevated temperature storage. The four vials with the mentioned solutions were stored at 55 °C for 4 weeks to investigate changes in color and chemical contents. All four vials were placed in a tailormade aluminum block placed on a temperature-controlled hotplate, with the thermocouple for the hotplate fitted in a hole in the block. Before and after 1 and 4 weeks of storage, electrolytes were photographed with a mobile phone camera to visualize color changes as well as characterized with ^1^H, ^13^C, ^19^F, and ^31^P NMR.

### NMR characterizations

Four hundred megahertz NMR was made using a JEOL RESONANCE ECZ400S spectrometer. 540 microlitres of the studied electrolyte solution were placed in an NMR glass tube in the argon-filled glovebox. Deuterated dimethyl sulfoxide (DMSO-d6) was placed in coaxial insert which were in turn placed in the NMR tube, which was then sealed with a lid and parafilm. NMR samples were measured within 2 days after being taken out of the glovebox. ^1^H spectra were referenced to DMSO-d5 traces at 2.500 ppm and ^13^C spectra were referenced to DMSO-d6 at 39.52 ppm^45^, whereas ^19^F spectra were referenced relative to the peak of NaPF_6_ at −72.7 ppm, as was done by Barnes et al.^22^. Spectra were normalized to the height of the largest peak.

### Battery cell assembly

Electrodes were provided by LiFeSiZE AB®, using commercial Prussian white powder Fennac produced by Altris AB® (12 mg cm^−2^) and hard carbon (7 mg cm^−2^), with an N/P ratio of 1.1 for the full cells. More specific details regarding composition etc. are found in the earlier publication^36^. Electrode disks were cut from foil sheets to 20 mm in diameter, before dried for 15 h in a vacuum oven at 170 °C, after which they were kept in an argon-filled glovebox. Pouch cells were assembled in the glovebox with 150 µL electrolyte, Dreamweaver gold separator, and 5 mm wide current collector tabs cut from thicker Al foil and placed behind each electrode disc.

### Galvanostatic cycling and Intermittent current interruption (ICI)

Full cells were cycled at 40 °C or 55 °C in a Novonix High Precision Charger System or Arbin Instruments battery testing system. The internal resistance of some cells was studied with ICI, for which an Arbin Instruments battery testing system was used. Some of the cells were equipped with reference electrodes based on preconditioned Prussian white, as in the previous publication^36^. Every 5 min, a 1 s pause with a recorded datapoint every 0.1 s was initiated. As the pause is initiated, there is a voltage drop linearly proportional to *t*^½^. The instantaneous voltage drop at *t* = 0 was extrapolated from the 10 data points within the pause, and using Ohm’s law, resistance was calculated for each pause^46^. The calculated resistance values were then averaged for each cycle.

For the cycling rate tests, discharge rate was increased from 0.2 C to 0.5 C, 1 C, 2 C, 4 C and 8 C with 10 cycles on each rate, whereas charge rate was kept at 0.2 C. The extended relaxation tests were carried out by adding 100 h pause after the 10th charge and 20th discharge, to investigate capacity loss at charged and discharged states, respectively.

### Pressure analysis

Pressure was measured during the three first 0.2 C galvanostatic cycles for the full cells assembled in PAT-Cell-Press from EL-cell® according to the method described in the earlier publication^36^. Tests were performed at 55 °C for the 0.35 M NaBOB–TEP, 0.35 M NaBOB–TEP + 3 wt% PES, and 1 M NaPF_6_ in EC:DEC electrolytes.

## Supplementary information

Supplementary information

## Data Availability

All relevant data are available from the authors upon request. Reza Younesi will be responsible for replying to this request. Galvanostatic cycle data is either in .csv (Novonix) or .xlsx file format (Arbin). NMR data is in the .jdf file format. This galvanostatic and NMR data is further summarized in Origin in .opju file format. Pressure analysis data is also in the .xlsx file format.
