# Publisher Correction: Effect of additives on the high-temperature performance of a sodium bis(oxalato)borate in triethyl phosphate electrolyte in sodium-ion batteries

**DOI:** 10.1038/s42004-025-01681-1

**Published:** 2025-09-09

**Authors:** Jonas Welch, Wessel W. A. van Ekeren, Jonas Mindemark, Reza Younesi

**Affiliations:** Department of Chemistry—Ångström Laboratory, Uppsala University, Uppsala, Sweden

**Keywords:** Batteries, Batteries

Correction to: *Communications Chemistry* 10.1038/s42004-025-01515-0, published online 26 April 2025

In the version of the article initially published, in the title, “trimethyl” should have read “triethyl” and has now been corrected in the HTML and PDF versions of the article.

