# Supplementary material for: Effect of additives on the high-temperature performance of a sodium bis(oxalato)borate in triethyl phosphate electrolyte in sodium-ion batteries
Source: Commun Chem. 2025 Apr 26;8:127. doi: 10.1038/s42004-025-01515-0 (PMC12033342; doi:10.1038/s42004-025-01515-0)
Supplement: Supplementary file 1 — Supplementary information [file 42004_2025_1515_MOESM1_ESM.pdf]

# Effect of additives on the high-temperature performance of a sodium bis(oxa-lato)borate in triethyl phosphate electrolyte in sodium-ion batteries

Jonas Welch[1], Wessel van Ekeren[1], Jonas Mindemark[1] and Reza Younesi[1]

[1]Department of Chemistry – Ångström Laboratory, Uppsala University, Box 538, 75121 Uppsala, Sweden

## $^{1}H$, $^{13}C$, $^{31}P$ and $^{19}F$ NMR spectra of electrolytes before and after storage at 55 °C

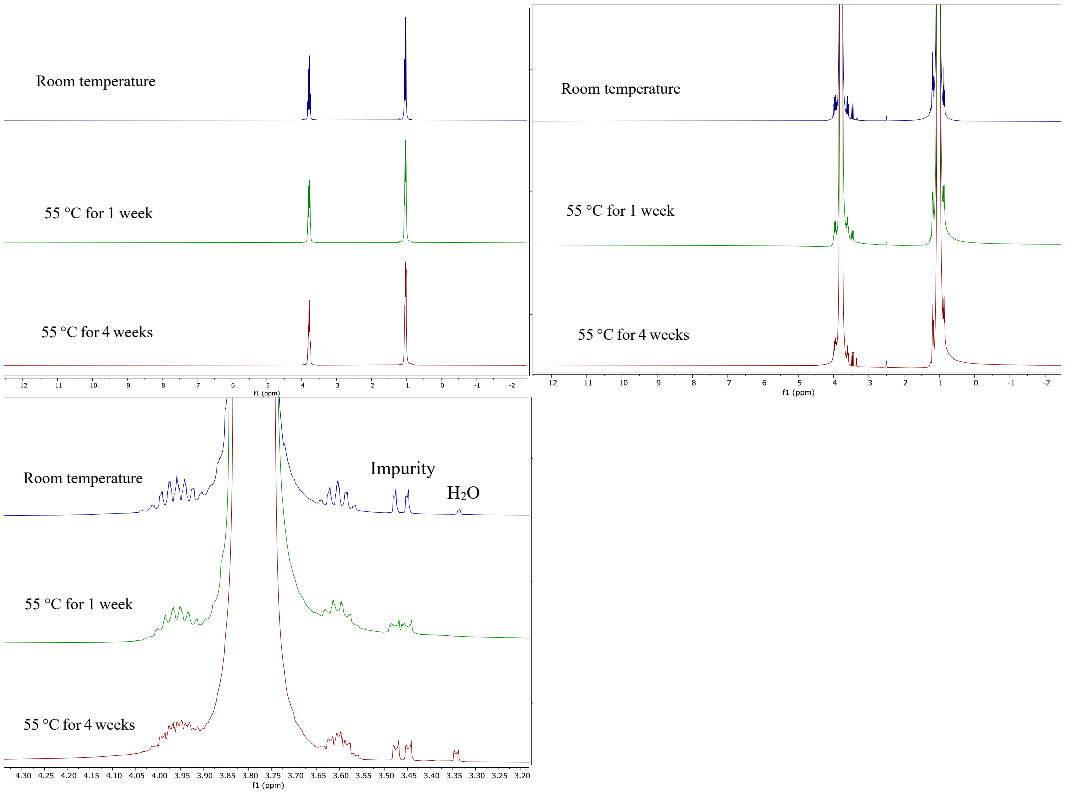


**Figure S1.** $^{1}H$ NMR of pristine 0.35 M NaBOB in TEP electrolyte and after 1 and 4 weeks of storage at 55 °C.

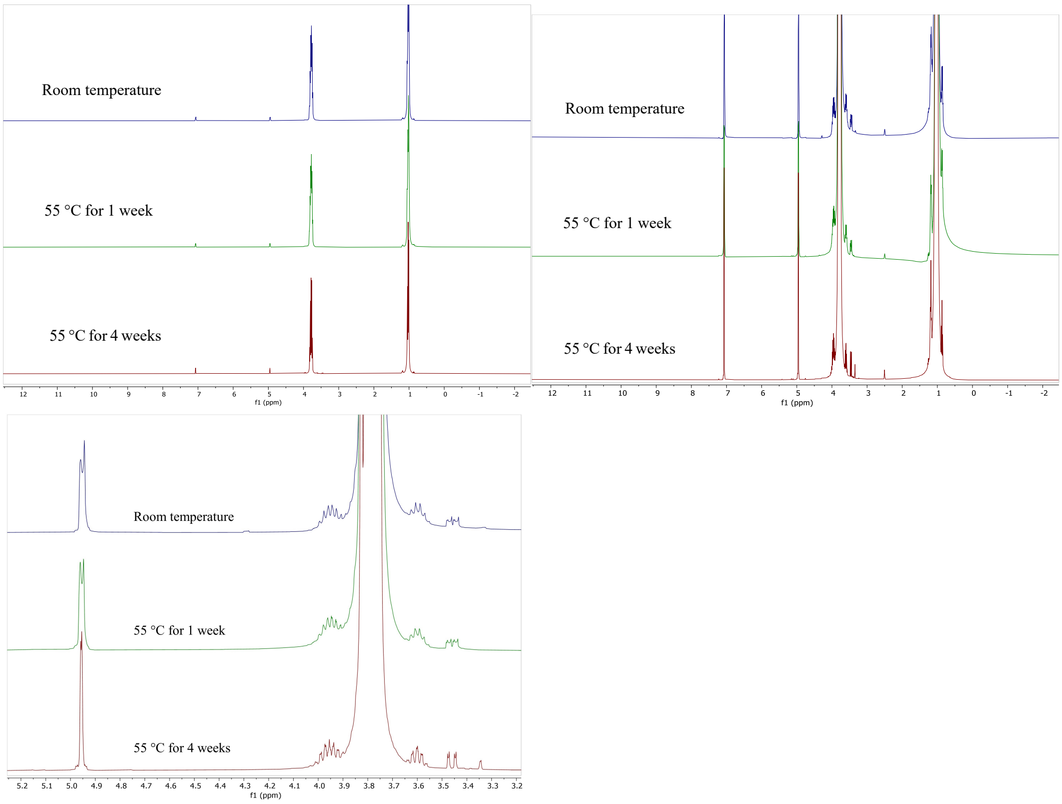


**Figure S2.** $^{1}H$ NMR of pristine 0.35 M NaBOB in TEP + 3 wt% PES electrolyte and after 1 and 4 weeks of storage at 55 °C.

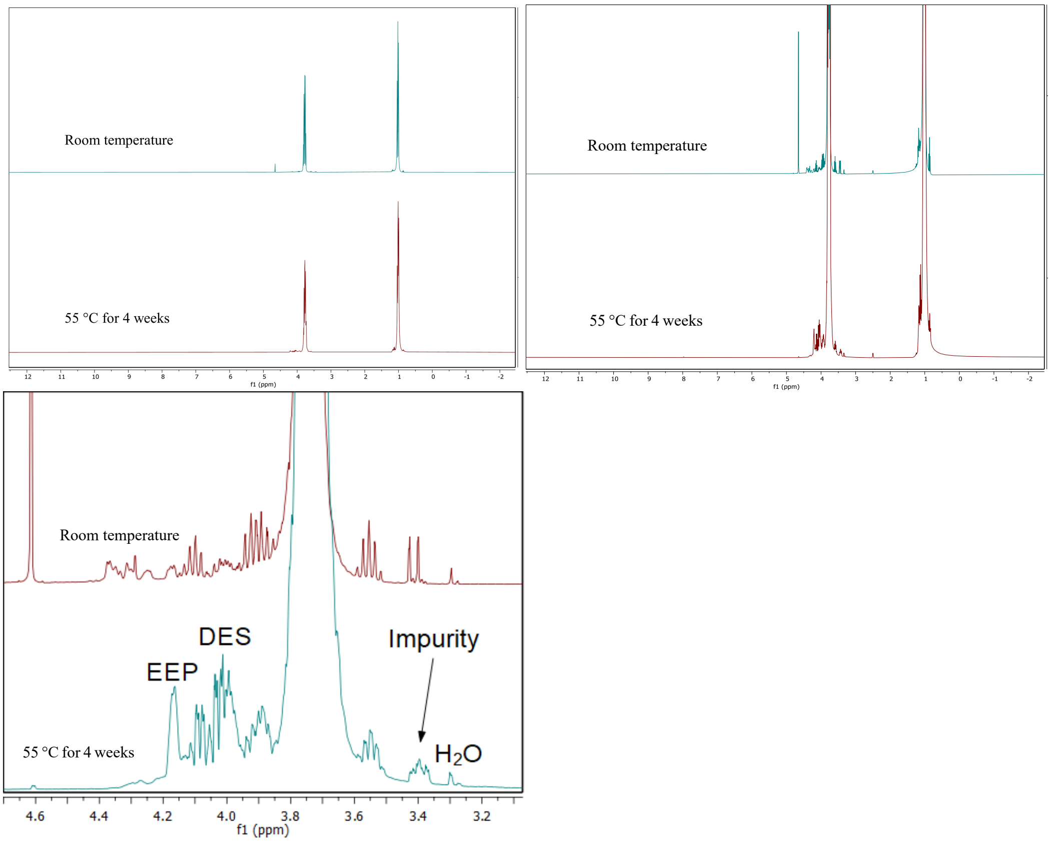


**Figure S3.** $^{1}H$ NMR of pristine 0.35 M NaBOB in TEP + 3 wt% DTD electrolyte and after 4 weeks of storage at 55 °C. Diethyl sulfate (DES) and ethyl ethylene phosphate (EEP) degradation products are marked in the figure.

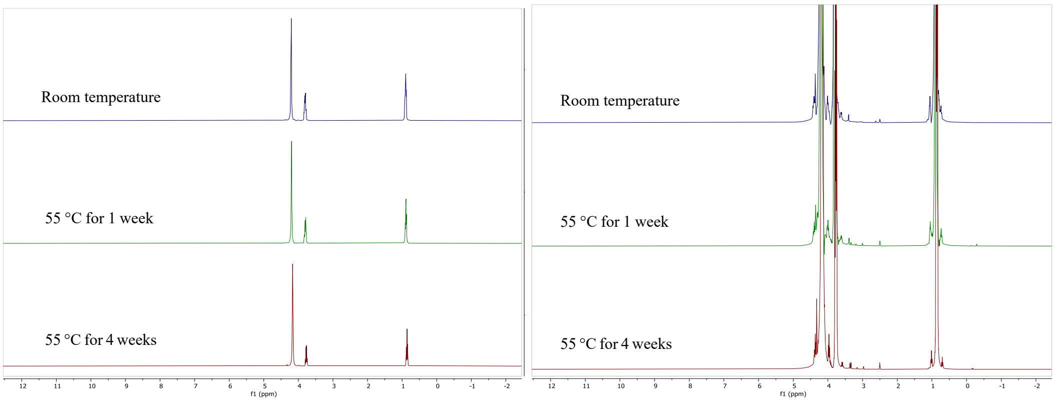


**Figure S4.** $^{1}H$ NMR of pristine 1 M $NaPF_6$ in EC:DEC electrolyte and after 1 and 4 weeks of storage at 55 °C.

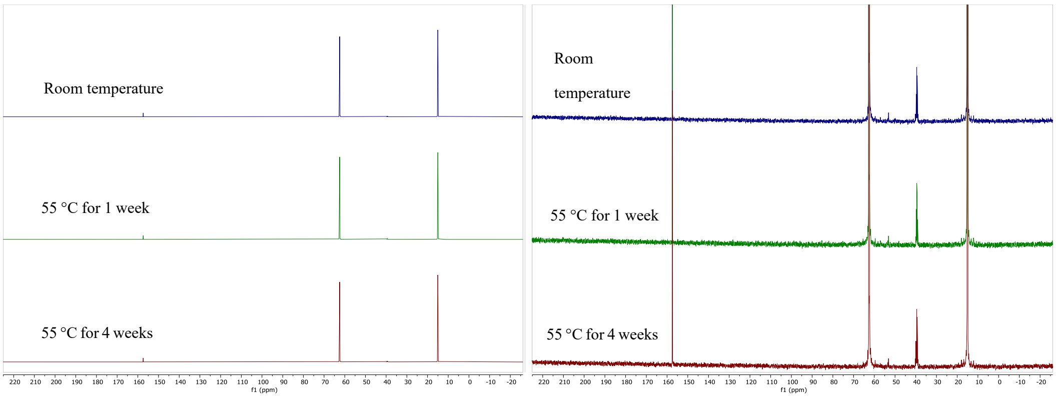


**Figure S5.** $^{13}C$ NMR of pristine 0.35 M NaBOB in TEP electrolyte and after 1 and 4 weeks of storage at 55 °C.

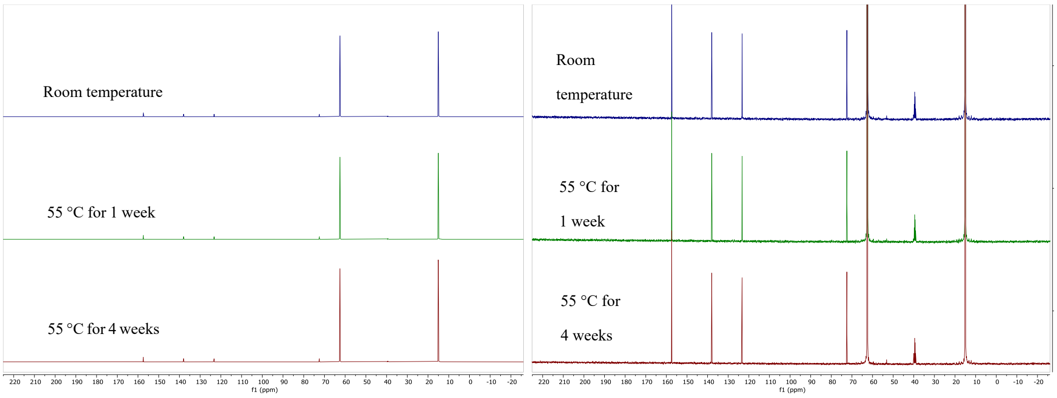


**Figure S6.** $^{13}C$ NMR of pristine 0.35 M NaBOB in TEP + 3 wt% PES electrolyte and after 1 and 4 weeks of storage at 55 °C.

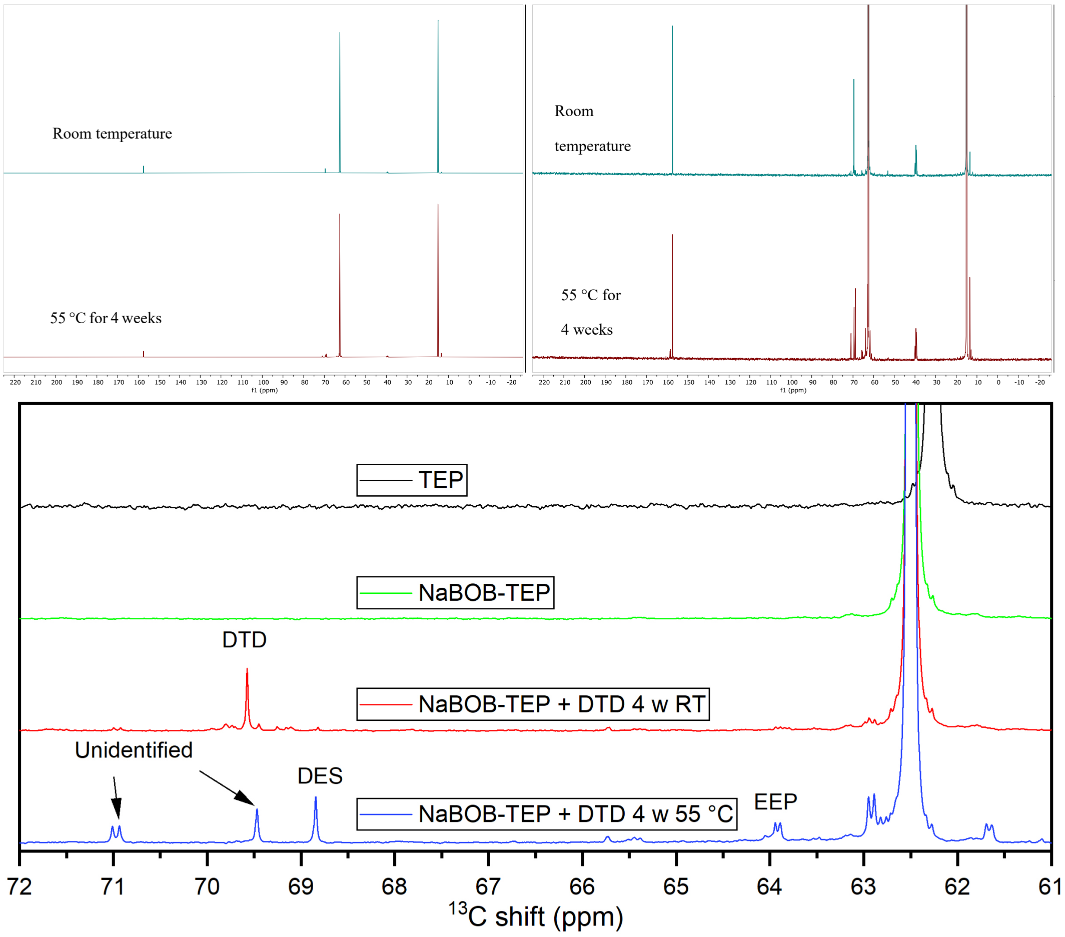


**Figure S7.** $^{13}$C NMR of pristine 0.35 M NaBOB in TEP + 3 wt% DTD electrolyte and after 1 and 4 weeks of storage at 55 °C. Diethyl sulfate (DES) and ethyl ethylene phosphate (EEP) degradation products are marked in the figure.

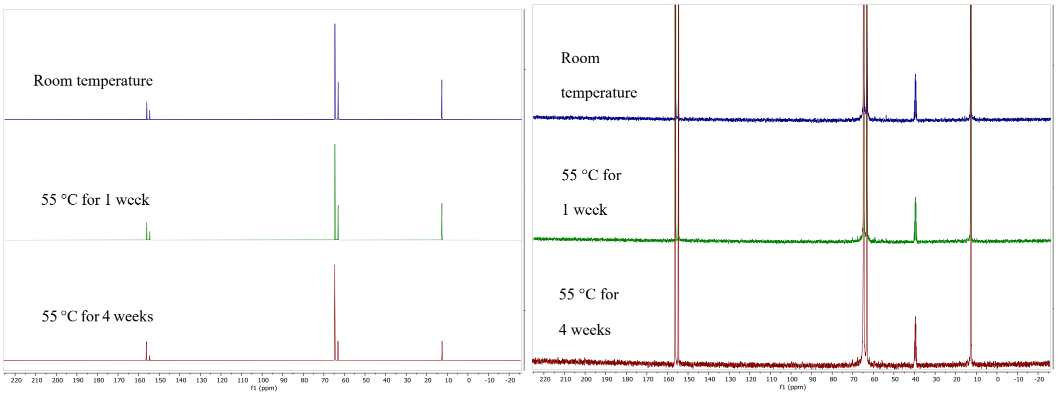


**Figure S8.** $^{13}$C NMR of pristine 1 M $NaPF_6$ in EC:DEC electrolyte and after 1 and 4 weeks of storage at 55 °C.

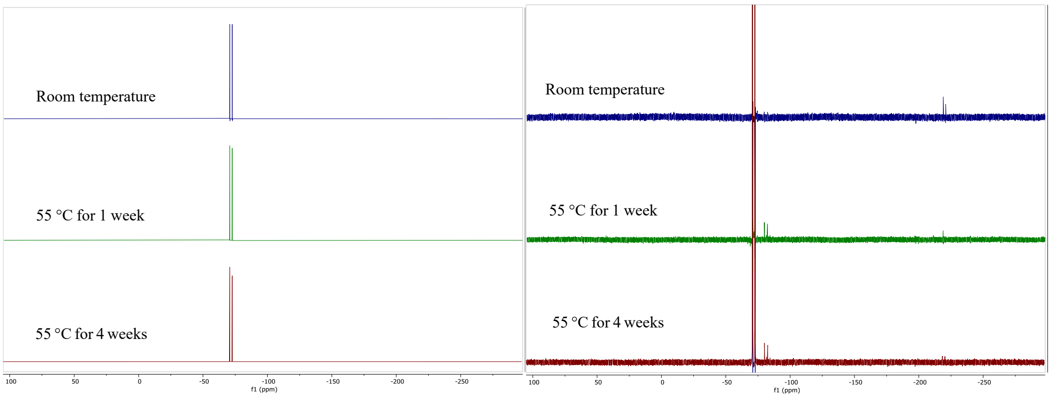


**Figure S9.** $^{19}F$ NMR of pristine 1 M $NaPF_6$ in EC:DEC electrolyte and after 1 and 4 weeks of storage at 55 °C.

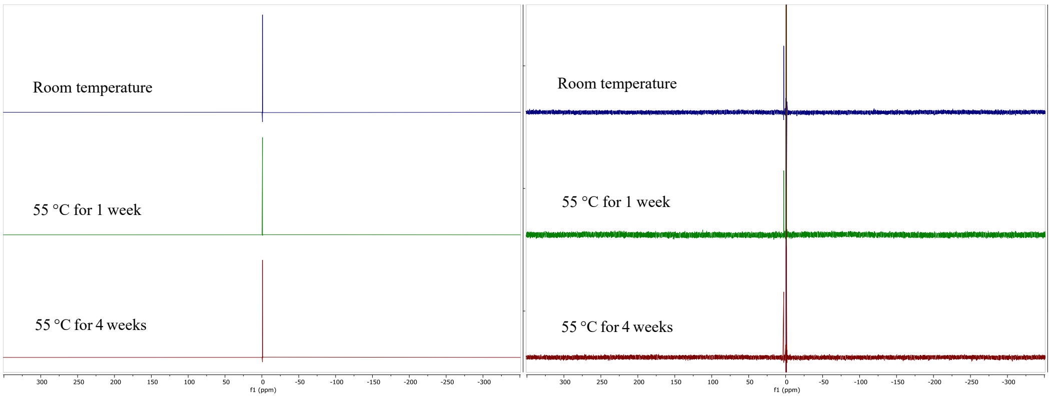


**Figure S10.** $^{31}P$ NMR of pristine 0.35 M NaBOB in TEP and electrolyte and after 1 and 4 weeks of storage at 55 °C.

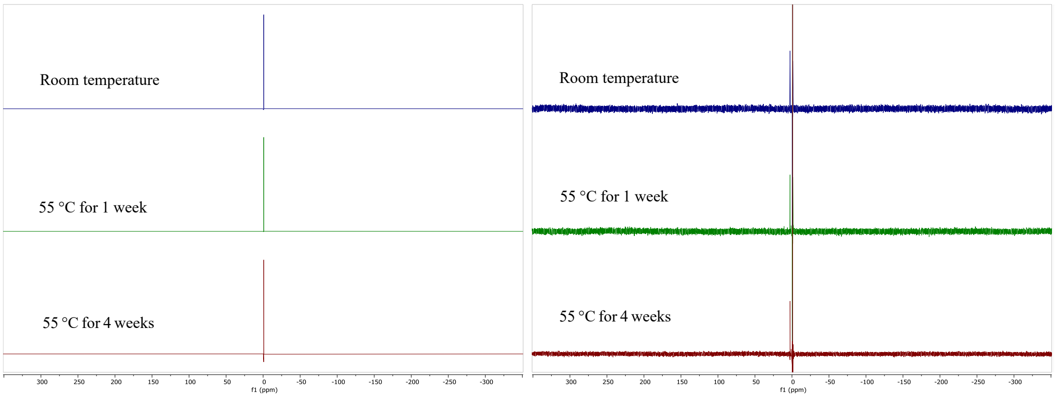


**Figure S11.** $^{31}P$ NMR of pristine 0.35 M NaBOB in TEP + 3 wt% PES electrolyte and after 1 and 4 weeks of storage at 55 °C.

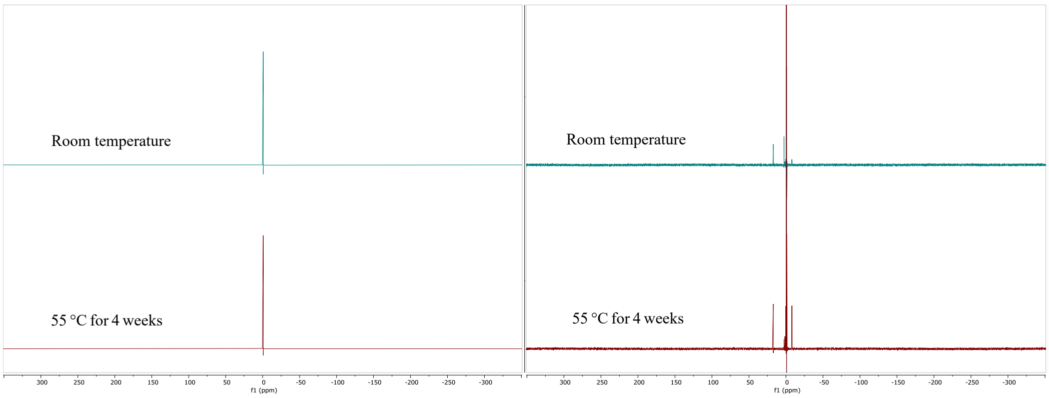


**Figure S12.** $^{31}P$ NMR of 0.35 M NaBOB in TEP + 3 wt% DTD

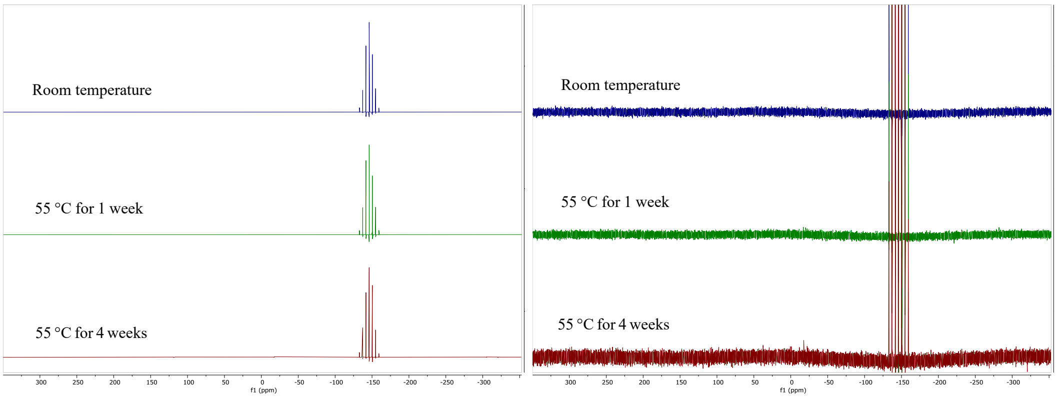


**Figure S13.** $^{31}P$ NMR of pristine 1 M $NaPF_6$ in EC:DEC electrolyte and after 1 and 4 weeks of storage at 55 °C.

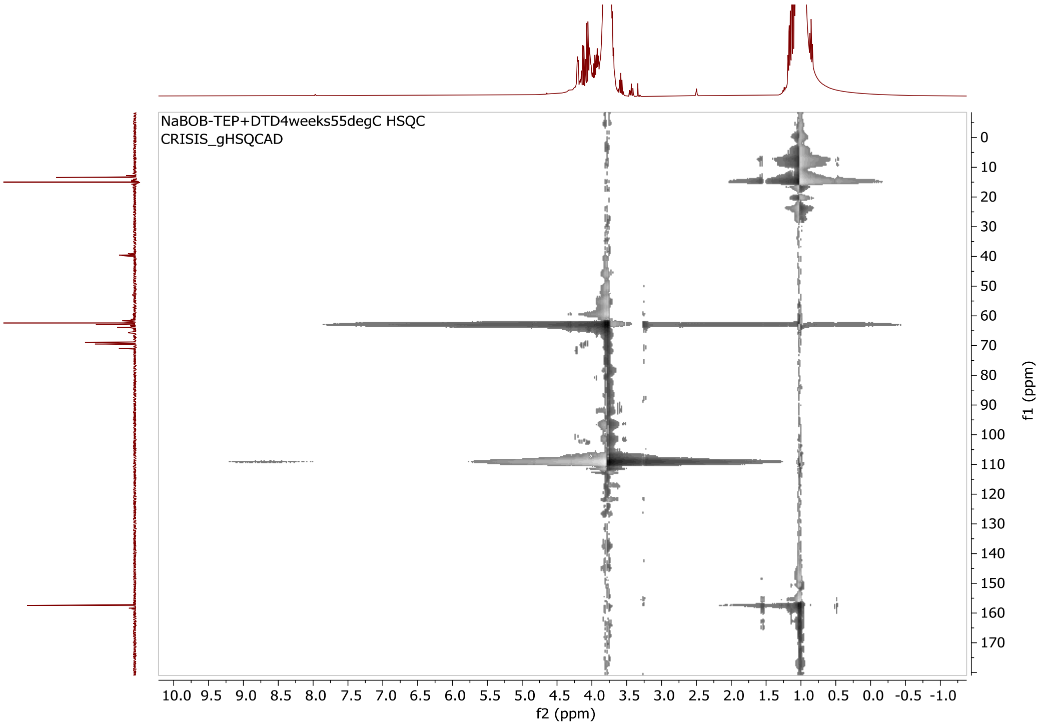


**Figure S14.** HSQC 2D NMR, showing correlations between $^{1}H$ and $^{13}C$ nuclei in the 0.35 M NaBOB-TEP + 3 wt% DTD that had been stored on a 55 °C hotplate for 4 weeks.

## Galvanostatic cycling and internal resistance measurements

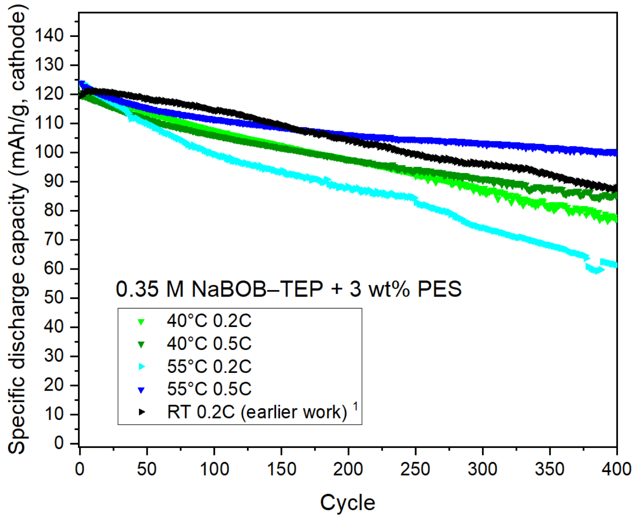


**Figure S15.** Galvanostatic cycling of Prussian white – hard carbon full cells using a 0.35 M NaBOB-TEP + 3 % PES electrolyte at 40 and 55 °C at 0.2C and 0.5C, including earlier published data [1] for a similar cell cycled at room temperature.

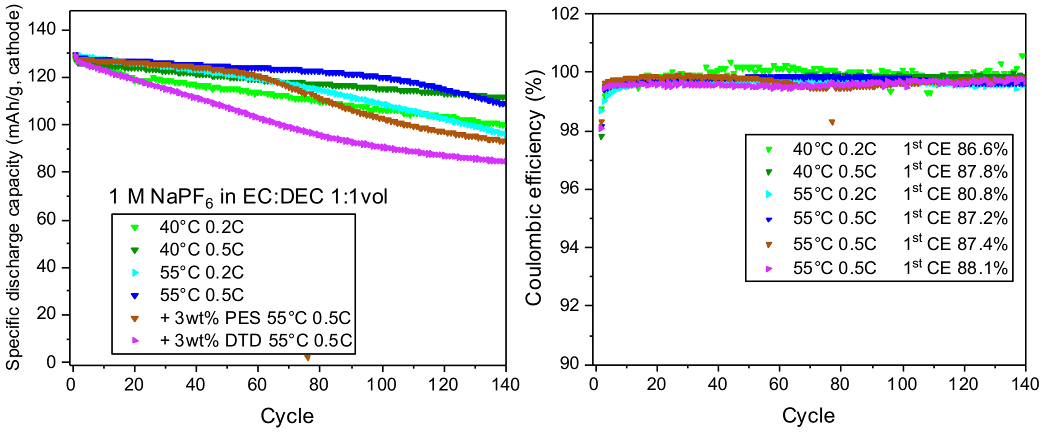


**Figure S16.** Galvanostatic cycling of Prussian white – hard carbon full cells using a 1 M $NaPF_6$ in EC:DEC electrolyte at 40 and 55 °C at 0.2C and 0.5C, and the same electrolyte with either 3 wt% PES or DTD additive in cells cycled at 0.5C.

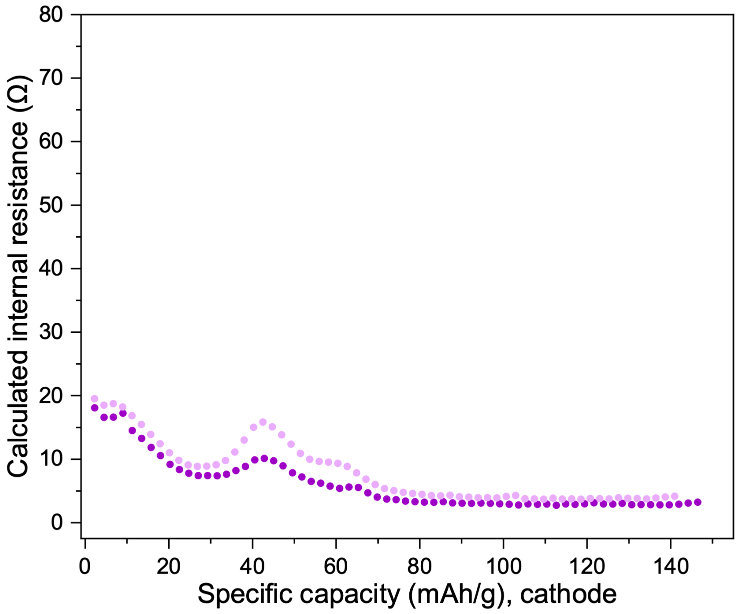


**Figure S17.** Internal resistance in Prussian white – Hard carbon full cells using a 1 M $NaPF_6$ in EC:DEC electrolyte measured with the ICI method during the first charge, when cycled at 55 °C.

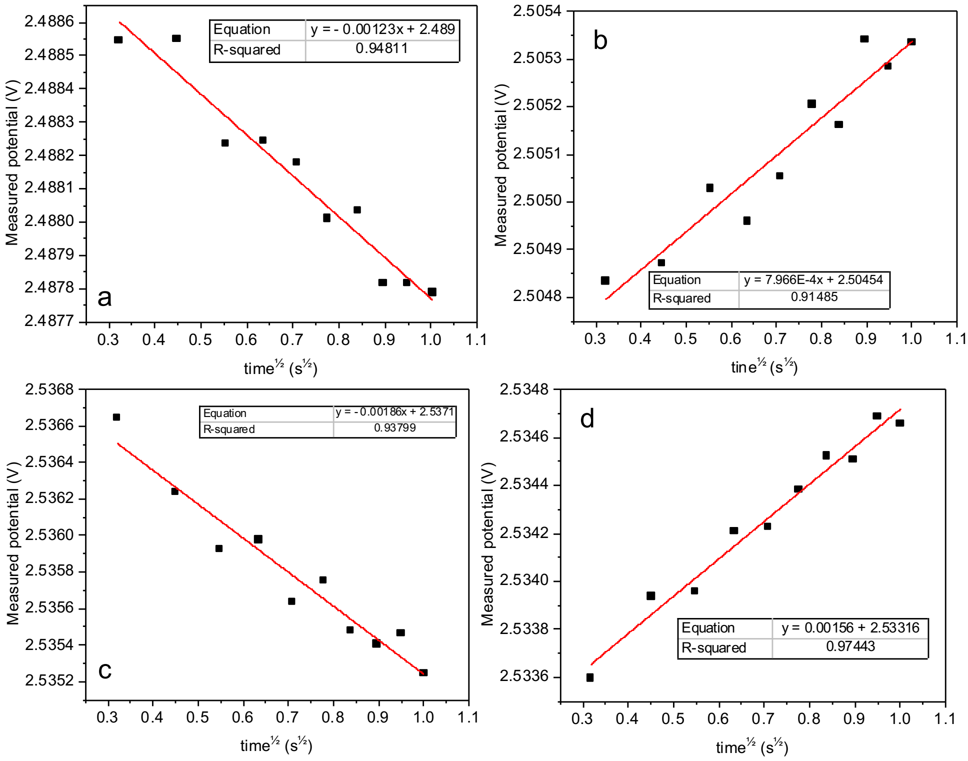


**Figure S18.** Resistance measurement of a 3-electrode cell cycling at 55 °C using 0.35 M NaBOB in TEP + 3 wt% PES electrolyte solution. Each figure depicts the 10 data points measured during one intermittent current interruption initiated when the cell voltage was close to 2.5 V. a) 20th charge, b) 20th discharge, c)120th charge and d) 120th discharge

## Pressure analysis

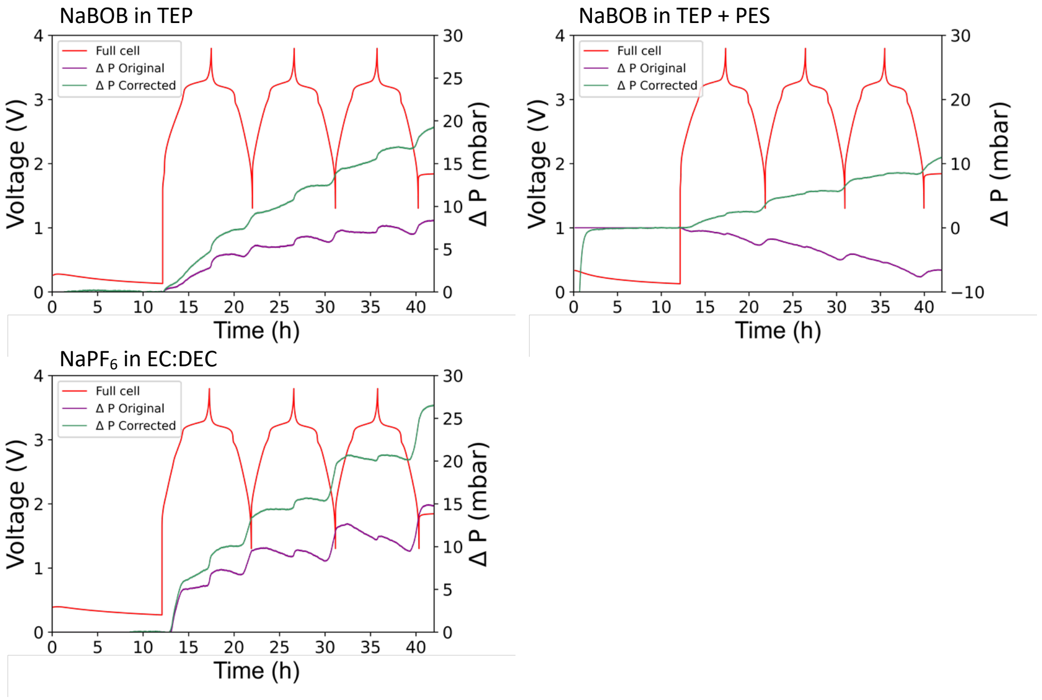


**Figure S19.** Original pressure data and data corrected for the measured leakage during the 2 hours directly before the initiation of galvanostatic cycling for EL cells containing Prussian white and hard carbon electrodes, cycling at 55 °C using electrolyte solutions of 0.35 M NaBOB in TEP (top left), 0.35 M NaBOB in TEP + 3 wt% PES (top right) and 1 M $NaPF_6$ in EC:DEC (bottom).

## Reference:


1 Welch, J. *et al.* Optimization of Sodium Bis(oxalato)borate (NaBOB) in Triethyl Phosphate (TEP) by Electrolyte Additives. *J Electrochem Soc* **169**, 120523, doi:10.1149/1945-7111/acaa5e (2022).
